# The efficacy of transcutaneous electrical nerve stimulation on the improvement of walking distance in patients with peripheral arterial disease with intermittent claudication: study protocol for a randomised controlled trial: the TENS-PAD study

**DOI:** 10.1186/s13063-017-1997-1

**Published:** 2017-08-10

**Authors:** Florent Besnier, Jean-Michel Sénard, Vincent Grémeaux, Mélanie Riédel, Damien Garrigues, Thibaut Guiraud, Marc Labrunée

**Affiliations:** 1 Institute of Metabolic and Cardiovascular Diseases (I2MC) team 8, Université de Toulouse National Institute of Health and Medical Research (INSERM), Toulouse, France; 2 Clinic of Saint-Orens, Cardiovascular and Pulmonary Rehabilitation Centre, Saint-Orens-de-Gameville, France; 3 Department of Clinical Pharmacology, Toulouse University Hospital, Toulouse, France; 4 Pôle Rééducation-Réadaptation and Plateforme d’Investigation Technologique INSERM CIC 1432, Dijon University Hospital, Dijon, France; 5 Department of Cardiovascular Rehabilitation, Reunion University Hospital, Réunion Island, France; 6 Department of Cardiovascular Rehabilitation, Toulouse University Hospital, Toulouse, France

**Keywords:** Peripheral arterial disease, Transcutaneous electrical nerve stimulation, Endothelial function, Physical activity

## Abstract

**Background:**

In patients with peripheral arterial disease (PAD), walking improvements are often limited by early pain onset due to vascular claudication. It would thus appear interesting to develop noninvasive therapeutic strategies, such as transcutaneous electrical nerve stimulation (TENS), to improve the participation of PAD patients in rehabilitation programmes, and thus improve their quality of life. Our team recently tested the efficacy of a single 45-min session of 10-Hz TENS prior to walking. TENS significantly delayed pain onset and increased the pain-free walking distance in patients with class-II PAD. We now seek to assess the efficacy of a chronic intervention that includes the daily use of TENS for 3 weeks (5 days a week) on walking distance in Leriche-Fontaine stage-II PAD patients.

**Methods/design:**

This is a prospective, double-blind, multicentre, randomised, placebo-controlled trial. One hundred subjects with unilateral PAD (Leriche-Fontaine stage II) will be randomised into two groups (1:1). For the experimental group (TENS group): the treatment will consist of stimulation of the affected leg (at a biphasic frequency of 10 Hz, with a pulse width of 200 μs, maximal intensity below the motor threshold) for 45 min per day, in the morning before the exercise rehabilitation programme, for 3 weeks, 5 days per week. For the control group (SHAM group): the placebo stimulation will be delivered according to the same modalities as for the TENS group but with a voltage level automatically falling to zero after 10 s of stimulation. First outcome: walking distance without pain. Secondary outcomes: transcutaneous oxygen pressure (TcPO_2_) measured during a Strandness exercise test, peak oxygen uptake (VO_2_ peak), endothelial function (EndoPAT®), Ankle-brachial Pressure Index, Body Mass Index, lipid profile (LDL-C, HDL-C, triglycerides), fasting glycaemia, HbA1c level, and the WELCH questionnaire.

**Discussion:**

TENS-PAD is the first randomised controlled trial that uses transcutaneous electrical therapy as an adjuvant technique to improve vascular function in the treatment of PAD. If the results are confirmed, this technique could be incorporated into the routine care in cardiovascular rehabilitation centers and used in the long term by patients to improve their walking capacity.

**Trial registration:**

ClinicalTrials.gov, ID: NCT02678403. Registered on 9 February 2016. Sponsor: Toulouse University Hospital.

**Electronic supplementary material:**

The online version of this article (doi:10.1186/s13063-017-1997-1) contains supplementary material, which is available to authorized users.

## Background

Peripheral arterial disease (PAD) is characterised by narrowing of the arteries feeding the lower limbs (subdiaphragmatic aorta, iliac arteries, lower-limb arteries) [1, 2]. PAD causes a loss of haemodynamic load, with or without clinical manifestations. The most reliable sign is the fall in the Ankle-brachial Pressure Index (ABI). The severity of the disease is assessed using the Leriche-Fontaine classification (Table 1).

**Table 1:** Leriche-Fontaine classification of peripheral arterial disease (PAD)

Stage 1	Asymptomatic PAD. Absence of one or several peripheral pulses with no functional repercussions
Stage 2	Symptomatic PAD. Intermittent claudication manifesting as pain on walking, signs of muscle ischaemia on effort. ‘Mild’ intermittent claudication (stage 2a) if the walking distance is greater than 200 m or ‘severe’ (stage 2b) if the walking distance is less than 200 m
Stage 3	Severe PAD pain in the lower limb appearing at rest or in decubitus, a sign of permanent tissue ischaemia
Stage 4	Presence of trophic disorders or necrosis in the lower limbs, such as ulcers or gangrene, and indication of severe ischaemia in most cases leading to amputation

The prevalence is estimated at 2.2% and 1.2% in men and women older than 55 years, respectively, and substantially increases with age [3] up to 40% for both sexes after age 80 years [4, 5] and is associated with an excess risk of cardiovascular death [6]. According to the international REduction of Atherothrombosis for Continued Health (REACH) registry [7], 50% of PAD patients have polyvascular disease. On average, half of all patients will die within the 5 years following diagnosis [8, 9].

Vascular rehabilitation is principally based on walking, on the therapeutic education of the patient and on adapted gymnastics (American College of Cardiology Foundation/American Heart Association (ACCF/AHA) guideline recommendations 2013) [10, 11]. This must be implemented in conjunction with medical treatment at the claudication stage (stage 2) before considering revascularisation [1, 10]. To optimise reconditioning to effort, it is recommended to alternate phases of exercise during which the patient must walk until the pain threshold is reached (pain occurs just before onset of the claudication) followed by a 10-min phase of passive recovery [12]. Several randomised studies have shown that physical exercise based on walking significantly improved walking distance and walking speed, as well as participation in physical activities [13–17]. A recent Cochrane meta-analysis [18] showed that in 1816 PAD patients supervised physical exercise on a treadmill improved walking distance in patients from 50 to 200%, maximal walking time by 4.51 min (95% CI 3.11–5.92) and the distance walked by 108.99 m (95% CI 38.20–179.78) compared with classical medical management without physical exercise. Nonetheless, physical exercise did not improve the ABI (mean difference 0.05, 95% CI 0.00–0.09).

Despite the undeniable benefits of walking, this approach is very often limited by pain, which is felt relatively early, leading patients to stop walking after a few minutes and to have to rest for 5 to 10 min until discomfort/pain ceases before starting again. In addition, given this peripheral limitation, exercise intensity, and thus demand on the cardiovascular system, is often insufficient to obtain the global impact of reconditioning to effort which, therefore, remains relatively ineffective.

It therefore seems pertinent to develop new techniques as an addition to the classical methods for physical reconditioning in these patients. By decreasing pain and improving comfort, and thus walking potential in PAD patients, it should be possible to reach high enough levels of energy expenditure to have an impact on both central and peripheral aspects of physical reconditioning.

The effects of electrical stimulation on the lower limbs in PAD patients have been studied by Loubser et al. who reported vasodilatation in the affected leg after a single 60-min session [19]. In this study, the authors used neuromuscular electrical stimulation (NMES) at a frequency of 2 Hz, which induced twitches of muscle contraction as well as sensory stimulation. In a fairly similar protocol, a slight improvement in pain-free walking distance was reported [20]. Transcutaneous electrical nerve stimulation (TENS) is another type of electrical programme, which, unlike NMES, only induces sensory stimulation and is mainly used for its analgesic effect. In PAD patients, there are no data concerning the vasodilatory effect of TENS. However, a pilot study on the efficacy of a single session of TENS to the legs in the PAD patients before they walked on a treadmill was recently conducted by our team [21]. In this study, 15 stage-II PAD patients underwent 45 min of TENS previous to a standardised walking exercise on a treadmill. The TENS sessions were carried out in different experimental conditions: 10 Hz, 80 Hz, SHAM (presence of electrodes without stimulation), or CON (without electrodes). The results for walking in the low-frequency TENS group (10 Hz) were better than those in the other groups (*p* < 0.0003). No adverse effect was reported. These results suggest that a single session of 10-Hz TENS just before the walking exercise increases both the pain threshold and the walking distance in stage-II PAD patients.

We therefore hypothesised that the use of TENS in PAD patients would improve walking distance via its impact on two aspects of vascular claudication (reduced vascular flow and pain). This therapeutic technique has, nonetheless, never been used in routine cardiovascular rehabilitation. Based on our experience with TENS, we now wish to evaluate the efficacy of this technique in a chronic setting including a 45-min daily session, 5 days a week for 3 weeks, associated with a physical reconditioning protocol classically used in these patients.

## Methods/design

### Study design

The TENS-PAD study will be a prospective, double-blind, multicentre, randomised, placebo-controlled trial. Outcomes will be assessed before and after 3 weeks of a cardiac rehabilitation programme. The study protocol has been developed based on the Standard Protocol Items: Recommendations for Interventional Trials (SPIRIT) guidelines (Additional file 1). The trial is founded by the ‘Programme Hospitalier de Recherche Clinique’ (PHRC) supported by the French Ministry of Health and was approved by the Sud-Ouest Outre-Mer Committee for the Protection of Personnes and the Agence Nationale de Sécurité du Médicament et des Produits de Santé (2015-A01534-45). The promotor of the study is the Centre Hospitalier Universitaire de Toulouse. The study was registered at ClinicalTrials.gov (ID: NCT02678403).

**Fig. 1:**
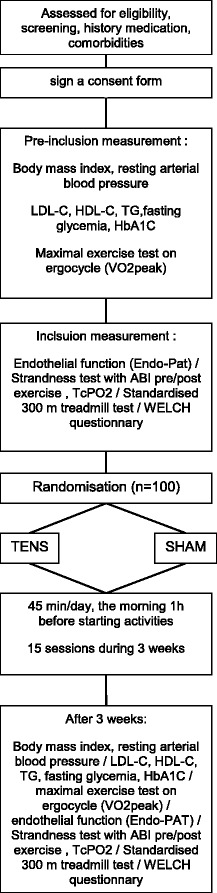
Flow chart for patient inclusion and follow-up, showing screening, inclusion measurements, randomisation, and tests at follow-up. ABI (Ankle-Brachial Index) ; PAD (Peripheral Artery Disease); TcPO2 (Transcutaneous Oxygen Pressure) ; TENS (Transcutaneous Electrical Nerve Stimulation) ; TG (Triglyceride) VO2peak (Oxygen consumption at peak exercise) ; WELCH (Walking Estimated Limitation Calculated by History)

**Fig. 2:**
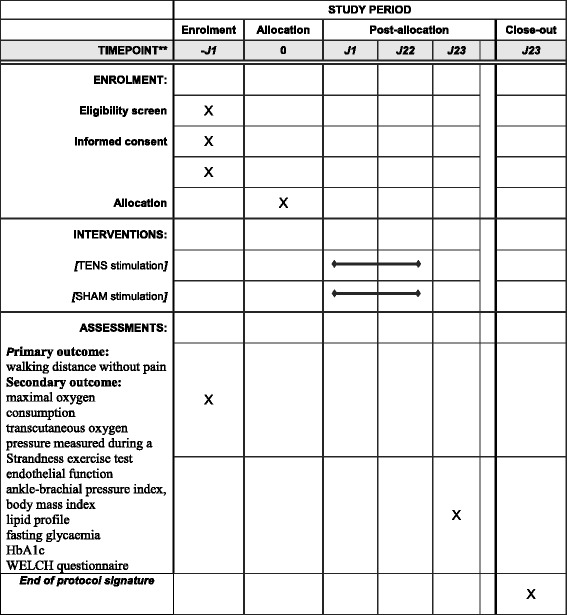
Standard Protocol Items: Recommendations for Interventional Trials (SPIRIT) figure

Figure 1 shows the flow chart indicating the patient inclusion, parameter measurement and follow-up process for the trial. The SPIRIT figure (Fig. 2) provides an overview of the study conduct, review, reporting, and interpretation, and the populated SPIRIT Checklist is presented in Additional file 1. The final report will follow the Consolidated Standards of Reporting Trials (CONSORT) guidelines, as well as the Template for Intervention Description and Replication (TIDieR).

### Participants

A total of 100 participants (aged from 18 to 85 years) admitted to a cardiovascular rehabilitation unit will be included in this study. Patients are eligible if (1) they present unilateral PAD (Leriche-Fontaine stage II) diagnosed by an angiologist who has previously performed a detailed arterial lesion description using an arterial Doppler ultrasound of the lower limbs, completed by microcirculatory assessment and showing an ABI ≤0.9, (2) they are able to take part in an outpatient rehabilitation programme, and (3) they are clinically stable. Participants will be excluded if they have (1) walking disorders related to orthopaedic or neuromuscular disease, (2) myopathy, (3) associated progressive disease causing deterioration in general health, or (4) an implanted pacemaker or defibrillator. All the inclusion and exclusion criteria are presented in Table 2.

**Table 2:** Inclusion and exclusion criteria

Inclusion criteria	Unilateral Leriche-Fontaine stage-II PAD, admitted to a cardiovascular rehabilitation unit Men or women Aged 18 to 85 years Able to take part in an outpatient rehabilitation programme Clinically stable Sedentary Provided informed consent to participate in the study
Exclusion criteria	Age >85 years Ward of court Walking disorders related to orthopaedic or neuromuscular disease Participation in a structured physical reconditioning programme in the month before the study Renal insufficiency requiring dialysis Known and documented myopathy Progressive cancer Associated progressive disease causing a deterioration in general health Participation in another research protocol Skin disorder making it impossible to use TENS Absolute contraindication to physical activity Presence of a pacemaker/defibrillator

*PAD* peripheral arterial disease, *TENS* transcutaneous electrical nerve stimulation

### Study procedure

Patients will be identified and recruited by the investigators in one of the four centres taking part in the study: le Centre Hospitalier Universitaire de Toulouse (France), la Clinique de Saint-Orens (France), le Centre Hospitalier Universitaire de Dijon (France), and le Centre Hospitalier Universitaire de la Réunion (French overseas territory). Patients will be informed about the study and asked for their participation. If they agree, they will be invited to sign the informed consent. After baseline assessment, they will be randomised into two groups (TENS versus SHAM). The duration of participation is 3 weeks. After the end of the cardiac rehabilitation programme all the participants will be re-evaluated.

In this trial, we will not modify the medical treatment that could interact with the walking capacity of the patient during the protocol, notably vasodilators, vasoconstrictors, antiplatelet drugs, and analgesics.

### Exercise intervention

#### ‘TENS’ group

TENS consists in delivering electrical stimulation to the affected leg via two channels and four electrodes placed on the triceps surae and the quadriceps muscles. Electrical stimulation characteristics will be: 10-Hz frequency, symmetrical compensated rectangular biphasic pulses (200 μs), maximal intensity just below the pain threshold, 45 min per day, in the morning 1 h before the exercise, 15 sessions spread over 3 weeks.

#### ‘SHAM’ group

Placebo stimulation will be delivered using the same modalities as in the TENS group, but with a current that automatically switches off after 5 s of stimulation.

### Outcomes

The primary outcome of the study will be the evolution of walking distance on a treadmill between before (D_0_) and after rehabilitation (D_21_), with or without TENS, measured in metres according to a standardised protocol (300-m test) [21].

The secondary outcomes of the study will be the maximal duration of walking, variation in TcPO_2_, the ABI value measured during the Strandness exercise test, variations in peak VO_2_, endothelial function (using the EndoPAT® system), cardiovascular risk factors (Body Mass Index, lipids (LDL-cholesterol, HDL-cholesterol, triglycerides), resting arterial blood pressure, glycaemic status (fasting glycaemia, glycosylated haemoglobin (HbA1c) level), and the scores obtained in the WELCH questionnaire [22].

### Blinding and randomisation

The study participants will be randomised into two balanced groups (1:1): (1) TENS and (2) SHAM. The randomisation will be stratified according to the four inclusion centers, and the two arms (TENS and SHAM) will be distributed in random-sized blocks (blocks of 2, 4 or 6). The method of randomisation used will be Ralloc on stata 9.2 S.E.

The assessor executing the measurements and the investigators will be blinded to group allocation. The statistician will be blinded until completion of the statistical analysis. Only the physiotherapists executing the cardiac rehabilitation programme will be aware of the assigned group of randomisation. Physiotherapists will not participate to the baseline or final measurements.

Participants will be blinded regarding the randomisation group to which they belong. The physiotherapists will install the TENS device and electrodes and will programme the TENS unit (pulse width, frequency, and intensity) according to the TENS or SHAM group (using a current that automatically switches off after a few seconds of stimulation).

### Measurements (before and after 3 weeks of the cardiovascular rehabilitation programme)

#### 300-m treadmill test

This walking test on a constant-intensity treadmill (300-m test) to measure walking distance (principal judgement criterion) is a procedure which is used for the rehabilitation of PAD patients and that has been described by our team to evaluate patients’ walking potential [21, 23]. For this test, the speed and the slope of the treadmill are initially adjusted in such a way to make claudication develop at a distance of 300 m. Five successive walking pre-tests (with periods of passive recovery of 5 min between tests) are necessary to obtain these treadmill settings with a baseline speed of X km/h and a baseline slope of X%. Once these parameters have been established, they will be kept constant for all of the pre- and post-treatment evaluations. This will allow us to determine the improvement in walking distance. During the test, the distance covered will be hidden from patients so as not to modify their performance. Each evaluation will consist of five successive tests with 5 min of passive recovery between the tests. The pre-test will take place at least 1 h before the initial test.

*The constant intensity effort test on a treadmill (Strandness exercise test)* will be supervised by a therapist in a room devoted to rehabilitation on a treadmill conducted according to the protocol described by Strandness [24]. The test starts by measuring ankle blood pressure at rest. The walking speed is constant (2 mph or 3.2 km/h) and the slope is fixed at 10%. This procedure, which has been validated and is classically used for the rehabilitation of PAD patients, allows a quantitative and reproducible evaluation of the haemodynamic repercussions related to PAD. The walking test is generally stopped when the subject can no longer continue (pain in the lower limbs). The test ends with the measurement of blood pressure at the ankle just after the exercise.

*The cardiorespiratory maximal effort test* will be supervised by a cardiologist in a room devoted to bicycle ergometer exercises. After a 3-min rest phase with the patient sitting on the apparatus and a 2-min warm-up phase at 20 W, the workload will be progressively increased by 15 W every minute until the maximal capacity of the patient has been reached according to the classical recommendations in the academic literature [25]. The imposed pedalling frequency will be between 60 and 70 rotations per minute (rpm). The exercise will be conducted until exhaustion of the subject, whatever the reason for stopping. The test will end with an active (2 min at 30 W) and then a passive recovery phase (4 min without load sitting on the apparatus). During this test, the subjects will be continuously monitored with a 12-lead electrocardiogram and by measuring arterial blood pressure in a relaxed arm every 2 min. Pulmonary gas exchange and the ventilatory responses will be measured continuously using an automated ‘breath-by-breath’ gas analysis system. Arterial oxygen saturation will be recorded throughout the maximal effort test using a pulse oximeter. The test can be stopped by the cardiologist in cases of cardiac intolerance to effort (severe arrhythmia, poorly controlled arterial hypertension, significant myocardial ischaemia, etc.). In such cases the patient will be excluded from the research protocol.

### Measurement of endothelial function (using the EndoPAT® system)

Plethysmography probes will be placed on the index fingers of both hands. Each probe includes a latex membrane which is inflated by a micro-compressor incorporated in the device, and a pressure close to diastolic arterial blood pressure is maintained. The probes thus fit the finger perfectly and measure variations in pulse-wave amplitude which reflects variations in capillary blood volume. The protocol consists in carrying out a 5-min ischaemia test of one of the two forearms by complete occlusion of the brachial artery by inflating a cuff to 60 mmHg above the systolic arterial blood pressure to a minimum of 200 mmHg. The amplitude of the pulse wave is recorded continuously on both sides for 15 min: 5 min before occlusion (baseline), during the 5 min of occlusion (ischaemia) and during the first 5 min post occlusion (hyperaemia). The RH-PAT (Reactive Hyperaemia-Peripheral Arterial Tonometry) Index is calculated automatically by the built-in software. The RH-PAT Index is the ratio between the mean pulse-wave amplitude, calculated between 90 and 120 s after releasing the occlusion, and mean pulse-wave amplitude during the 210 s preceding the occlusion. In order to eliminate confounding systemic neurovegetative vasomotor effects, this index is normalised with regard to the contralateral side.

*The WELCH (Walking Estimated Limitation Calculated by History) questionnaire* is a self-administered questionnaire validated in French and specific to PAD [22]. Via four questions (‘duration of slow walking’ , ‘duration of moderate walking’ , ‘duration of fast walking’ , ‘comparison of one’s walking speed with that of one’s entourage’), it evaluates the symptoms of the claudication in everyday life. It takes about 5 min to complete the questionnaire.

### Sample size calculation and statistical analysis

According to our pilot study [21], the expected benefit is an improvement of 25% in walking distance without pain in the TENS group compared with the placebo group at 3 weeks. Considering a mean value of the principal criterion of 300 m in patients in the SHAM group, with a standard deviation of 105 m common to both groups, a power of 90% with a bilateral hypothesis and an alpha risk of 5%, a difference of 25% in the walking distance between the two groups at the end of the study (that is to say a mean value of 300 m versus a mean value of 375 m), it is necessary to include 42 subjects per group. This number will be increased to 50 subjects per group (100 subjects altogether) to take into account an estimated 15% premature exit from the study.

Analysis of objective differences will be based on an analysis of covariance (ANCOVA) of the evolution of judgement criteria (adjusted for their initial value) rather than a simple comparison of values at the end of the follow-up. These ANCOVA models should increase the power of analyses concerning the above data [26]. After a description of the initial characteristics of the included subjects (for the whole population and by group), and after verification of the initial comparability of the two groups (numbers, percentages, mean ± standard deviation, or median and range), evaluation of the efficacy of the intervention will be made as a single analysis, on an intention-to-treat basis (according to the group attributed by randomisation), with a global alpha risk of 5%, a bilateral formulation of the statistical tests, normality of distribution and the homogeneity of variances will be evaluated graphically and by the Shapiro-Wilk and the Fisher-Snedecor normality tests. In cases of non-normal distributions, we will attempt to normalise data using appropriate transformations.

Analysis of the of principal judgement criterion: the evolution of the claudication distance between the start and the end of the study in the two intervention group (TENS versus SHAM) will be compared using ANCOVA adjusted for initial walking distance.

Analyses of the secondary judgement criteria: according to the same principles as the principal criterion, an ANCOVA according to the intervention group (TENS versus SHAM) adjusted for the initial value of the judgement criterion will be used for each of the criteria.

## Discussion

Validating the efficacy of TENS as an adjuvant technique for physical exercise is an important opportunity to improve walking capacity in patients with vascular claudication.

It is well known that PAD patients have low-level physical activity; only 3500 to 4500 steps per day on average, which is far below World Health Organisation recommendations (10,000 steps per day) [27–30]. It is interesting to note that TENS has been already studied in healthy subjects. Several studies have reported that low-frequency TENS improves the perfusion of the superficial tissues, measured by enhanced laser Doppler imaging, by up to 40% [31–33]. In a PAD patient, one very recent case study reported encouraging results on the improvement in ABI after using this technique [34]. The protocol used was as follows: 45 min per session, twice a day, for 3 months. The ABI of the affected leg increased from 0.63 to 0.71 after 3 months (then to 0.80 1 month later at a follow-up consultation), while the ABI in the leg without TENS did not change. The authors concluded that prospective, randomised controlled studies are necessary to better understand the effects of low-frequency TENS on the improvement in muscle perfusion in PAD patients.

Our team studied the effects on TENS on muscle sympathetic nerve activity (MSNA) in chronic heart failure (CHF) [35]. According to the results of our study, both TENS and neuromuscular electrostimulation (NMES) are able to reduce MSNA compare to sham stimulation (63.5 ± 3.5 versus 69.7 ± 3.1 bursts/min, *p* < 0.01 after TENS and 51.6 ± 3.3 versus 56.7 ± 3.3 bursts/min, *p* < 0.01 after NMES), suggesting that TENS to the lower limbs could inhibit sympathetic outflow directed to the legs in CHF patients and could be able to reduce vasoconstriction. In PAD, oxidative stress and reduced blood flow to working muscles induce the augmented blood pressure response to exercise caused by an exaggerated exercise pressor reflex (PR) [36–38]. The PR is a neurological reflex that increases sympathetic nervous system activity, constricts arterioles and increases blood pressure. It is well known that TENS treatment releases endogenous opioids in the central nervous system and produces analgesia [39–41]. Furthermore, it has been shown in previous animal studies that the stimulation of opioid receptors of group-III and -IV afferent endings contributes to attenuating the exaggerated exercise PR observed in rats with ligated femoral arteries [38, 42, 43]. Thus, according to our hypothesis, TENS could be a new technique to reduce sympathetic nerve activity, the PR, and to induce a neuromodulation of the vascular tonus in PAD in addition to its traditional direct analgesic effects. This easy-to-use therapeutic tool could prove to be extremely interesting as it is easy to transport for use at home so that patients can use it to complement daily walking exercise. In the long term this could be useful in discouraging a sedentary lifestyle and will hopefully guarantee a higher level of physical activity and thus have an impact on secondary prevention after PAD.

### Study limitations

Current guidelines recommend a supervised exercise programme for 3 months. Our 3-week trial, therefore, is relatively short and the results will not be optimal. That is why all patients should continue their exercises at home to maintain and to ameliorate their walking distance. Nevertheless, our clinical experience shows that improvements in walking distance without pain are visible very early with several sessions a day of treadmill exercise. That leads us to believe that 3 weeks are not optimal but sufficient to see some benefits in rehabilitation, whatever the group (TENS or SHAM).

Furthermore, the TENS device is probably able to perform both analgesic modulation and neuromodulation. However, other specific studies are needed to explore the local effect of TENS on vasodilatation and whether or not blocking the painful stimulus is beneficial.

### Trial status

Recruitment for the study has not yet started.

## Additional file

Additional file 1: SPIRIT 2013 Checklist: Recommended items to address in a clinical trial protocol and related documents. (DOC 114 kb)

## Abbreviations

ABI: Ankle-brachial Pressure Index
ACCF/AHA: American College of Cardiology Foundation/American Heart Association
CIC: Clinical Investigation Center
CONSORT: Consolidated Standards of Reporting Trials
I2MC: Institute of Metabolic and Cardiovascular Diseases
INSERM: Institute of Health and Medical Research
MSNA: Muscle sympathetic nerve activity
NMES: Neuromuscular electrical stimulation
PAD: Peripheral arterial disease
PR: Pressor reflex
REACH: REduction of Atherothrombosis for Continued Health
RH-PAT: Reactive Hyperaemia-Peripheral Arterial Tonometry
SPIRIT: Standard Protocol Items: Recommendations for Interventional Trials
TcPO_2_: Transcutaneous oxygen pressure
TENS: Transcutaneous electrical nerve stimulation
VO_2_peak: Peak oxygen consumption
WELCH: Walking Estimated Limitation Calculated by History
