# Supplementary material for: The efficacy of transcutaneous electrical nerve stimulation on the improvement of walking distance in patients with peripheral arterial disease with intermittent claudication: study protocol for a randomised controlled trial: the TENS-PAD study
Source: Trials. 2017 Aug 10;18:373. doi: 10.1186/s13063-017-1997-1 (PMC5553808; doi:10.1186/s13063-017-1997-1)
Supplement: Additional file 1: — SPIRIT 2013 Checklist: Recommended items to address in a clinical trial protocol and related documents. (DOC 114 kb) [file 13063_2017_1997_MOESM1_ESM.doc]

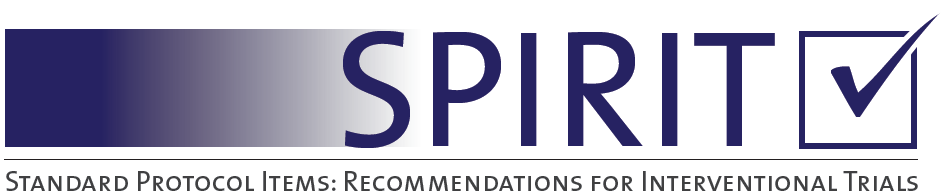


SPIRIT 2013 Checklist: Recommended items to address in a clinical trial protocol and related documents*

| Section/item | Description |  |
| --- | --- | --- |
| **Administrative information** | | |
| Title | Efficacy of transcutaneous electrical nerve stimulation in the improvement of walking distance in patients with peripheral artery disease with intermittent claudication: a randomized controlled trial.  TENS-PAD study |  |
| Trial registration | ClinicalTrials.gov NCT02678403 (received: 9 february 2016) |  |
| Protocol version | Version 3.0 (27.11.2015) |  |
| Funding | Programme Hospitalier de Recherche Clinique Interrégional’ (PHRCi) supported by the Ministry of Health |  |
| Roles and responsibilities | ML, TG, FB, MR conceived the research. Principal investigator: ML  Statistical analysis and fund raising: ML. Drafting and revision of the manuscript: FB, ML, JMS, VG, TG. All authors read and approved the final manuscript. |  |
|  | Trial sponsor : CHU de Toulouse, Hôtel-Dieu, 2 rue Viguerie,  31052 Toulouse cedex 9.  drci.toulouse@chu-toulouse.fr |  |
|  | Coordinating Centre: Dr Marc Labrunée (chief investigator) - PH- MPR, SSR Cardiovasculaire – CHU de Toulouse, labrunee.m@chu-toulouse.fr  Data management Team: Methdological and Data Center, Dr. LEPAGE Benoît, Service d'épidémiologie CHU Toulouse, Faculté de Médecine, 37, allées Jules Guesde 31073 Toulouse cedex, benoit.lepage@univ-tlse3.fr |  |
| Introduction |  |  |
| Background and rationale | Walking is indicated for patients with peripheral artery disease (PAD) to increase the pain-free walking distance. Nevertheless, improvements are often limited by early pain onset due to vascular claudication. It appears interesting to develop non-invasive therapeutic strategies, such as transcutaneous electrical nerve stimulation (TENS), to improve the participation of PAD patients in rehabilitation programmes, and thus improve their quality of life. Our team recently tested the efficacy of one single 45-minute session of 10 Hz TENS prior to walking. TENS significantly delayed pain onset and increased the pain-free walking distance in patients with class II PAD. We now seek to assess the efficacy of an intervention that includes the daily use of TENS for 3 weeks (5 days a week) on walking distance in PAD (Leriche-Fontaine stage II). |  |
| Objectives | The principal objective of the TENS-PAD study is to evaluate the efficacy of 3 weeks of TENS on walking distance in patients with PAD at the vascular claudication stage. The principal judgement criterion of the study is the evolution of walking distance on a treadmill before and after rehabilitation measured in metres according to our standardized protocol (300m test). The secondary objectives of the study aim to evaluate the efficacy of TENS on global physical aptitude (VO_2 peak_), endothelial function (measured using the EndoPAT® system), the blood lipids and quality of life (WELCH questionnaire). |  |
| Trial design | Design: prospective multicentre study / randomized placebo controlled trial / double blinding. 100 subjects with unilateral Peripheral Artery Disease (Leriche-Fontaine stage II) will be randomized into 2 groups (1:1). Experimental group (TENS group) and Control group (group SHAM). |  |
| Methods: Participants, interventions, and outcomes | | |
| Study setting | Patients will be recruited in one of the four rehabilitation centres taking part in the study: the Toulouse University Hospital, the Cardiovascular rehabilitation center of Saint Orens, the Dijon University Hospital, La Réunion University Hospital. |  |
| Eligibility criteria | Inclusion Criteria: Unilateral Leriche stage II PAD, admitted to cardiovascular Rehabilitation Unit, Men or women, Aged 18 to 85 years, Able to take part in an out-patient rehabilitation programme, Clinically stable, Sedentary, Provided informed consent to participate in the study. Exclusion Criteria: Age > 85 years, Ward of court, Walking disorders related to orthopaedic or neuromuscular disease, Participation in a structured physical reconditioning programme in the month before the study, Renal insufficiency requiring dialysis, Known and documented myopathy, Progressive cancer, Associated progressive disease causing a deterioration in general health, Participation in another research protocol, Skin disorder making it impossible to use TENS, Absolute contra-indication to physical activity, Presence of pacemaker / defibrillator. |  |
| Interventions | The study participants will be randomized into two balanced groups (1:1): (1) TENS; (2) SHAM. The randomization will be stratified according to the 4 inclusion centres, and the two arms (TENS and SHAM) will be distributed in random-sized blocks (blocks of 2, 4 or 6). The duration of participation is 21 days, that is to say 15 sessions.  Exercise intervention:  « TENS » Group: TENS consists in delivering electrical stimulation to the affected leg using a current delivered via two channels and four electrodes placed on the triceps surae and the quadriceps (10 Hz, symmetrical compensated rectangular biphasic pulses, 200 µs, maximal intensity just below the pain threshold, 45 minutes per day, in the morning 1h before the exercise, 15 sessions spread over 3 weeks).  Group « SHAM »: Placebo stimulation will be delivered using the same modalities as for the TENS group, but with a current that automatically switches off after a few seconds of stimulation. |  |
| Outcomes | Primary outcome: walking distance without pain (in meters) on treadmill. Secondary outcome: maximal oxygen consumption (VO2peak), transcutaneous oxygen pressure (TcPO2) measured during a Strandness exercise test, endothelial function (measured using the EndoPAT® system), ankle-brachial pressure index, body mass index, lipid profile (LDL-C, HDL-C, Triglycerides), fasting glycaemia, HbA1c, score to the WELCH questionnaire (Walking Estimated Limitation Calculated by History). |  |
| Participant timeline | Enrolment will take two years.  The duration of participation of each patient is 21 days, that is to say 15 sessions. Outcomes measures are assessed before and after 15 sessions. |  |
| Sample size | The expected benefit is an improvement of 25% in walking distance without pain in the TENS group compared with the placebo group at 3 weeks. Considering a mean value of the principal criterion of 300 m in patients of the « placebo » group, with a standard deviation of 105 m common to both groups, a power of 90% with a bilateral hypothesis and an alpha risk of 5%, a difference of 25% in the walking distance between the two groups at the end of the study (that is to say a mean value of 300m *versus* a mean value of 375m), it is necessary to include 42 subjects per group. This number will be increased to 50 subjects per group (100 subjects altogether) to take into account premature exit from the study of 15% of subjects. |  |
| Recruitment | Each patient addressed in the Cardiovascular rehabilitation Centers of the study with unilateral PAD will be screened to verify eligibility criterias. |  |
| **Methods: Assignment of interventions (for controlled trials)** | | |
| Allocation: |  |  |
| Sequence generation | The randomization will be stratified according to the 4 inclusion centres, and the two arms (TENS and SHAM) will be distributed in random-sized blocks (blocks of 2, 4 or 6). The method of randomization used is Ralloc on stata 9.2 S.E. |  |
| Implementation | each co-investigator of each centers will verify the eligibility of patients and then, randomisation will be generated (Dr. Benoit Lepage, data manager), the data manager will return to the physiologists the assignation group to interventions. |  |
| Blinding (masking) | After assignment to interventions, participant, data assessor, co-investigators, and data analysts will be blinded. |  |
| **Methods: Data collection, management, and analysis** | | |
| Data collection methods | All data will be collected in a Case Report Form (CRF). |  |
| Data management | Data will be simple data entered, and data validation will take place according to the procedures set out in the data management and data validation plan. Prior to any statistical analysis, all variables will be checked for missing values. Any questions regarding the data will go back to the data manager. Descriptive statistics will be calculated for all variables and distributional assumptions checked. |  |
| Statistical methods | Analysis of objective differences was based on an analysis of covariance (Ancova) of the evolution of judgement criteria (adjusted for their initial value) rather than a simple comparison of values at the end of the follow-up. These Ancova models should increase the power of analyses concerning the above data. After a description of the initial characteristics of the included subjects (for the whole population and by group), and after verification of the initial comparability of the two groups (numbers, percentages, mean ± standard deviation or median and range), evaluation of the efficacy of the intervention will be done as a single analysis, on an intention-to-treat basis (according to the group attributed by randomization), with a global alpha risk = 5 %, a bilateral formulation of the statistical tests, normality of distribution and the homogeneity of variances will be evaluated graphically and by the Shapiro-Wilk and F-Snedecor normality tests. In cases of non-normal distributions, we will attempt to normalize them using appropriate transformations.  Analysis of the of principal judgement criterion: the evolution of the claudication distance between the start and the end of the study in the two intervention group (TENS vs SHAM) will be compared using analysis of covariance adjusted for initial walking distance.  Analyses of the secondary judgement criteria: according to the same principles as the principal criterion, an analysis of covariance according to the intervention group (TENS vs SHAM) adjusted for the initial value of the judgement criterion will be done for each of the following criteria: evolution between the start and end of the study: maximal duration of walking, variation in tcPO2 and ankle-brachial index measured during the Strandness test, variations in peak VO2, in endothelial function (using the EndoPAT® system), cardiovascular risk factors (body mass index, lipids (LDL Cholesterol, HDL Cholesterol, Triglycerides), resting arterial pressure, glycaemic status (fasting glycaemia, HbA1C), and in the scores obtained in the WELCH questionnaire. |  |
| **Methods: Monitoring** | | |
| Data monitoring | To ensure high quality data, an independent Clinical Research Associate (CRA) will check all the data (identifiers removed, specifics study codes to identify each patient, writing informed consent, all the datas collected in the CRF). |  |
| Harms | All adverse events are recorded in the relevant CRF and to the chief investigator and adverse effects during TENS sessions (pain and muscle soreness) are recorded in the relevant case report form by the research physiologist. Serious adverse events are recorded by the Chief Investigator and declare to the sponsor and to the vigilance unit of the sponsor (Dr. Pascale OLIVIER-ABBAL; pascale.olivier1@univ-tlse3.fr). |  |
| Ethics and dissemination | | |
| Research ethics approval | The study was approved by the Research Ethics Committees of the French South-West and Overseas (2015-A01534-45). |  |
| Protocol amendments | Protocol amendments will be agreed and approved by the Research Ethics Committees of the French South-West and Overseas. |  |
| Consent or assent | All eligible patients are identified by investigators and approached with written information about the trial before to start any examens. If the patient chooses to participate in this trial, the first research visit is organised where written informed consent is obtained. |  |
| Confidentiality | All the datas will be entered in the Unit of Methodology with all direct patient identifiers removed, patients will be identified by specifics study codes. All data will be stored in a secured locked computer. |  |
| Declaration of interests | All the authors declare that they have no competing interests. |  |
| Access to data | Only members of the Clinical Research team will have access to patient records. |  |
| Ancillary and post-trial care | Provisions, if any, for ancillary and post-trial care, and for compensation to those who suffer harm from trial participation |  |
| Dissemination policy | Results of the study will be send to the sponsor and to the Research Ethic Committee. All dissemination of findings will be previously referred and approved by the chief investigator. |  |
| Appendices |  |  |
| Informed consent materials | Before to participate, each patient will received an informed consent and a writing information about the study (description, objective, methods to collect the data, course of events, expected results). |  |

*It is strongly recommended that this checklist be read in conjunction with the SPIRIT 2013 Explanation & Elaboration for important clarification on the items. Amendments to the protocol should be tracked and dated. The SPIRIT checklist is copyrighted by the SPIRIT Group under the Creative Commons “[Attribution-NonCommercial-NoDerivs 3.0 Unported](http://www.creativecommons.org/licenses/by-nc-nd/3.0/)” license.
